# Structural and physicochemical characteristics of starches from sorghum varieties with varying amylose content

**DOI:** 10.1002/fsn3.4245

**Published:** 2024-08-20

**Authors:** Longping Yang, Lei Li, Feng Jiang, Qiaoling Zhang, Fan Yang, Yilin Wang, Zhenyu Zhao, Qingfan Ren, Li Wang

**Affiliations:** ^1^ China Kweichow Moutai (Group) Distillery Co., Ltd., Key Laboratory of Characteristic Sorghum Renhuai Guizhou China; ^2^ Kweichow Moutai Co., Ltd. Renhuai Guizhou China; ^3^ Kweichow Moutai (Group) Hongyingzi Agricultural Science and Technology Development Co., Ltd. Renhuai Guizhou China

**Keywords:** amylose, physicochemical properties, sorghum starch, structural properties

## Abstract

Six sorghum varieties containing different amylose were selected to investigate the structural and physicochemical characteristics of starches and endosperm texture of grains. The results showed that the outer layer endosperm of sorghum grain changed from waxy to corneous, and its starch granules were more compactly packed and exhibited the spindle‐shaped holes with the increase of amylose content. Higher amylose starch granules exhibited fewer and smaller micropores on the surface and were more likely to deform and agglomerate into larger amorphous particles after heated. Amylose content of sorghum starches was negatively correlated to granule size, relative crystallinity, and the proportion of the long branch chains (DP = 25–36 and > 36), whereas positively correlated to the proportion of the short branch chains (DP = 6–12 and 13–24). Amylose content had negative correlations with *T*_*o*_, *T*_*p*_, Δ*H*, PV, and SDS (*p* < .05), positive corrections with FV, SB, and RDS (*p* < .05), and no correlations with *T*_*c*_, HPV, BD, and RS. It could be concluded that amylose content affected the endosperm texture of sorghum grain and had strong correlations with structural and physicochemical properties of sorghum starch. These findings may help identify uses for these sorghum varieties in baijiu production.

## Graphical abstract

Grain sorghum (*Sorghum bicolor* L. Moench) is an essential raw material for baijiu production. Amylose content affected the endosperm texture of sorghum grain and had strong correlations with structural and physicochemical properties of sorghum starch. These findings may help identify uses for these sorghum varieties in baijiu production.

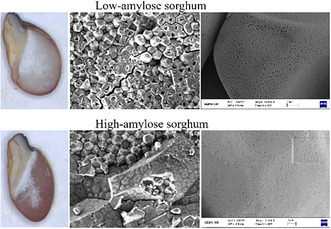

## 1 INTRODUCTION

Grain sorghum (*Sorghum bicolor* L. Moench) is an essential raw material for baijiu production. Starch is the most important composition of sorghum grain, and its structure and physicochemical properties are important factors in fermentation performance of sorghum varieties, and then affect the yield, flavor, and functionality of baijiu (Wang et al., 2018). Xu et al. (2021) reported that the sorghum varieties with higher total starch and amylopectin content could produce higher ethanol content. The starch structure may have played a major role in the fermentation performance of sorghum as demonstrated by Li et al. (2021), who showed that the crystalline structures of starch could influence the flavor and brewing efficiency of baijiu by affecting the amount and structures of leached starch. Wu et al. (2007) found that the bioconversion rate of sorghum grains would be adversely affected by high viscosity and high gelatinization temperature of starch. Moreover, Wang and Wu (2010) reported that starch digestibility was a key factor affecting the ethanol fermentation efficiency of sorghum grains. Ezeogu et al. (2005) found that the vitreousness of sorghum grains could affect starch digestibility. Yan et al. (2011) evaluated the fermentation performance of 25 sorghum varieties and found that waxy sorghums for ethanol production showed higher starch digestibility and fermentation efficiency. Therefore, endosperm texture and starch properties of sorghum grains could provide an important basis for manufacturers to select proper raw materials to further improve the yield and quality of baijiu.

Some studies have shown that the functional properties of starches from different individual varieties depend upon their morphological characteristics, crystallization, and the ratio of amylose to amylopectin (Buléon et al., 2007; Yoon et al., 2012). In addition, Zhou et al. (2015) found that wheat starch with high‐amylose content had high resistance to the cooking. Du et al. (2020) found that the amylose content had significant influence on the physicochemical properties of sago starch. The previous achievements have shown that amylose content played an important role in influencing starch properties. Nevertheless, the starch particle morphology, crystalline structure, and gelatinization properties of sorghum starches showed remarkable differences from other cereal starches (Jing et al., 2023). Besides, starch from different sorghum varieties also exhibited different physicochemical characteristics and processing properties. Singh et al. (2010) reported that 15 Indian sorghum cultivars contained 11.2–28.5% amylose and showed significant differences in physicochemical properties of starches and endosperm texture. Trust et al. (2001) analyzed the grain structure and starch properties of 10 sorghum varieties and found that amylose content ranged from 21.5% to 29.9% and was significantly negatively correlated with grain floury endosperm texture. Waxy sorghum grains contain very low‐amylose content; however, normal sorghum grains that are vitreous and corneous contain high‐amylose content (Choi et al., 2004). Zhang et al. (2023) studied gel properties of four Chinese sorghum hybrids containing different amylose and found that amylose and amylopectin ratios of sorghum starches could significantly affect retrograde properties. Sang et al. (2008) investigated the structure and functionality of sorghum starches differing in amylose content, but these starches were isolated from only three sorghum hybrids. Previous studies have revealed that amylose content was associated with the starch properties and endosperm texture of sorghum, but the results may be varied because of different sorghum varieties. Although sorghum is widely used in baijiu production, there are relatively few studies on researching the starch properties of sorghum varieties for baijiu production. Besides, the relationships between amylose content and sorghum starch properties were not clear.

This paper analyzed the starch properties and endosperm architecture of six sorghum varieties which were commonly used in the brewing industry and had a wide variation in amylose content, and investigated the correlations between amylose content and physicochemical properties of sorghum starch. The research results could help baijiu brewers find applications for these sorghum varieties with targeted brewing quality.

## 2 MATERIALS AND METHODS

### 2.1 Materials

Six sorghum samples with different amylose contents were used for this study. One Chinese sorghum variety (Hongyingzi 1619) was provided by Kweichow Moutai (Group) Hongyingzi Agricultural Science and Technology Development Co., Ltd., Guizhou, China. Five new variant strains (G5, G69, G72, G41, G42) were obtained from Hongyingzi 1619 variety through field selections for five consecutive generations by Henan Academy of Agricultural Sciences, Henan, China, and Kweichow Moutai Co., Ltd., Guizhou, China. The six sorghum samples (G5, Hongyingzi 1619, G69, G72, G41, G42) coded as S1, S2, S3, S4, S5, and S6, respectively. Hongyingzi 1619 variety is a conventional sorghum cultivar for Maotai‐flavor liquor production and is cultivated extensively in southwestern China. All varieties are red sorghum. All grain sorghums were planted in Guizhou, China, and grown in the 2020–2021 season.

### 2.2 Methods

#### 2.2.1 Starch extraction

Sorghum starch was isolated on the basis of Ni et al. (2022). Briefly, sorghum grain samples were steeped in 0.25% NaOH at 4°C for 24 h, and then the suspension was passed through 149 μm and 37 μm mesh sieves. The filtrate was centrifuged at 3000 *g* for 10 min, the sediment was mixed with MilliQ water and neutralized with acid chloride solution, and then centrifuged as above. This process was repeated thrice. Then, the starches were dried in oven at 40°C for 24 h. The starch–water mixtures were heated at 60°C, 70°C for 30 min, and centrifuged at 4000 *g* for 5 min, and the cooked starch samples were collected for further analyses.

#### 2.2.2 Kernel composition

Protein, starch, and lipid contents of sorghum grains were determined by GB 5009–2016 method. The tannin and amylose contents of sorghum grains were determined according to the method of Ebadi et al. (2005) and GB 7648–1987, respectively.

#### 2.2.3 Morphology and structure

Several mature seeds were hand sectioned. Grain endosperm texture was observed using a stereo microscope (SZX91T, Mingzi, Shanghai, China) as described by Ali et al. (2019). The morphology of the starch granules was observed using a scanning electron microscope (SEM) (TM‐1000, Hitachi, Tokyo, Japan). Amylopectin chain‐length distribution of sorghum starches was determined using a high‐performance anion‐exchange chromatography (HPAEC) (Dionex ICS‐5000^+^, Sunnyvale, CA, USA) according to a previous publication (Kong et al., 2008). The particle size was determined using a laser diffraction particle size analyzer (Mastersizer LS13320, Beckman Coulter, CA, USA). The crystalline characteristics of sorghum starches were measured using an X‐ray diffractometer (XRD) (X'Pert PRO, PANalytical, Almelo, the Netherlands), and the degree of relative crystallinity was calculated according to a previous publication (Kim & Huber, 2010).

#### 2.2.4 Gelatinization properties

Gelatinization properties of sorghum starches were analyzed using a Differential Scanning Calorimeter (DSC) (Q100; TA Instruments, New Castle, DE, USA). Starch samples (2 mg) were mixed with 6 μL of MilliQ water. The pan was equilibrated at room temperature for 2 h and then scanned at 10°C/min from 30°C to 110°C. The onset (*T*_*o*_), peak (*T*_*p*_), conclusion (*T*_*c*_) temperature, and enthalpy (*∆H*) of gelatinization were calculated automatically.

#### 2.2.5 Pasting properties

The peak viscosity (PV), hot paste viscosity (HPV), final viscosity (FV), breakdown (BD), and setback (SB) of sorghum starches were evaluated using a Rapid Visco Analyzer (RVA) (Model 4500, Perten Instrument, Hägersten, Sweden). Starch samples (3 g) were dispersed in MilliQ water (25 g). The programmed heating and cooling cycle was used according to the method described by Kong et al. (2015).

#### 2.2.6 In vitro digestion

Starch samples were dispersed in sodium acetate buffer. The obtained mixtures underwent heating at 95°C for 30 min, and the starch solution then was mixed with pancreas α‐amylase and amyloglucosidase. After incubating for 20, 120 min at 37°C, the filtrate was centrifuged at 4000 *g* for 15 min and the glucose content was determined using a Dionex ICS‐5000^+^ HPAEC system. The rapidly digestible starch (RDS), slowly digestible starch (SDS), and resistant starch (RS) were calculated by combining the glucose released at 20 and 120 min.

### 2.3 Statistical analysis

The data reported were an average of triplicate observations and were compared using t‐test or ANOVA to determine the significance of differences. Correlation analyses were determined with Pearson correlation coefficients.

## 3 RESULTS AND DISCUSSION

### 3.1 Proximate composition

The main chemical compositions of six sorghum varieties are summarized in Table 1. Starches isolated from all sorghum varieties were white in color. A significant variation of amylose content (0.2%–21.3%) was obtained from all sorghum samples. The six sorghum samples could be classified into three groups according to the amylose content: low, medium, and high. The low group (<10% amylose) included the S1 and S2 (0.2% and 7.5% amylose, respectively), medium group (10–20% amylose) included the S3 and S4 (17.9% and 19.2% amylose, respectively), and high group (>20% amylose) included the S5 and S6 (20.8% and 21.3% amylose, respectively). The six sorghum starches had a range of total starch content from 73.4% to 74.1%, which is slightly higher than that of previously reported sorghum. Sang et al. (2008) reported 68.4–71.4% of starch contents in three sorghum hybrids, while Xu et al. (2021) reported 64.2–69.0% of starch contents in five Australian sorghum varieties and 71.7% in a Chinese variety. However, there were no statistically significant differences in the total starch, protein, lipid, and tannin contents of the six sorghum grains.

**TABLE 1:** Main chemical components of different sorghum grains.

Sample	Starch (%)	Amylose (%)	Protein (%)	Tannin (%)	Lipid (%)
S1	73.4 ± 0.11a	0.2 ± 0.11a	10.6 ± 0.08a	1.4 ± 0.02a	4.5 ± 0.13a
S2	73.5 ± 0.08a	7.5 ± 0.08b	10.9 ± 0.11a	1.3 ± 0.01a	4.9 ± 0.09a
S3	73.5 ± 0.09a	17.9 ± 0.03c	11.1 ± 0.12a	1.2 ± 0.05a	4.7 ± 0.08a
S4	73.6 ± 0.12a	19.2 ± 0.05d	10.9 ± 0.09a	1.2 ± 0.06a	4.8 ± 0.11a
S5	74.0 ± 0.05a	20.8 ± 0.05e	10.4 ± 0.02a	1.4 ± 0.02a	4.4 ± 0.12a
S6	74.1 ± 0.06a	21.3 ± 0.08e	10.5 ± 0.05a	1.5 ± 0.03a	4.7 ± 0.07a

*Note*: Different lowercase letters on the shoulders of the same indicator indicate significant differences (*p* < .05).

### 3.2 Structural properties

#### 3.2.1 Grain endosperm texture

**FIGURE 1:**
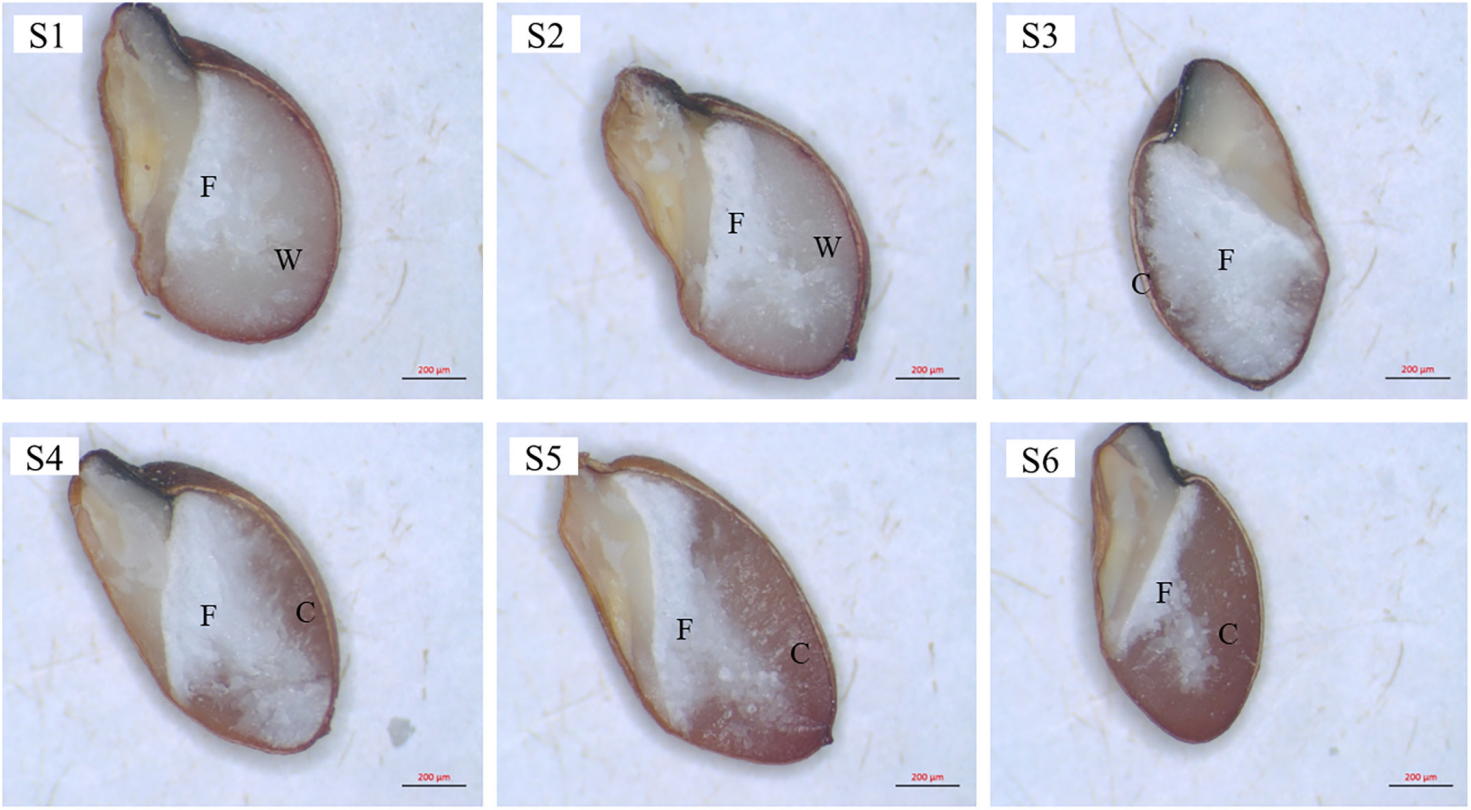
Grain endosperm texture of six sorghum grains. F refers to floury endosperm, W refers to waxy endosperm, and C refers to corneous endosperm.

By observing the endosperm texture of sorghum grains, it was found that the differences in the endosperm of six sorghum grains were apparent (Figure 1). The center endosperm of all sorghum grains was floury (F). The outer layer endosperm of S1 and S2 was waxy (W) and nearly indistinguishable. The outer layer endosperm of S3, S4, S5, and S6 was vitreous and corneous (C), whereas S3 and S4 had a much smaller proportion of corneous endosperm compared to S5 and S6. This result is in agreement with the findings of Trust et al. (2001), who found that endosperm texture and amylose content of sorghum grain had a significant correlation.

**FIGURE 2:**
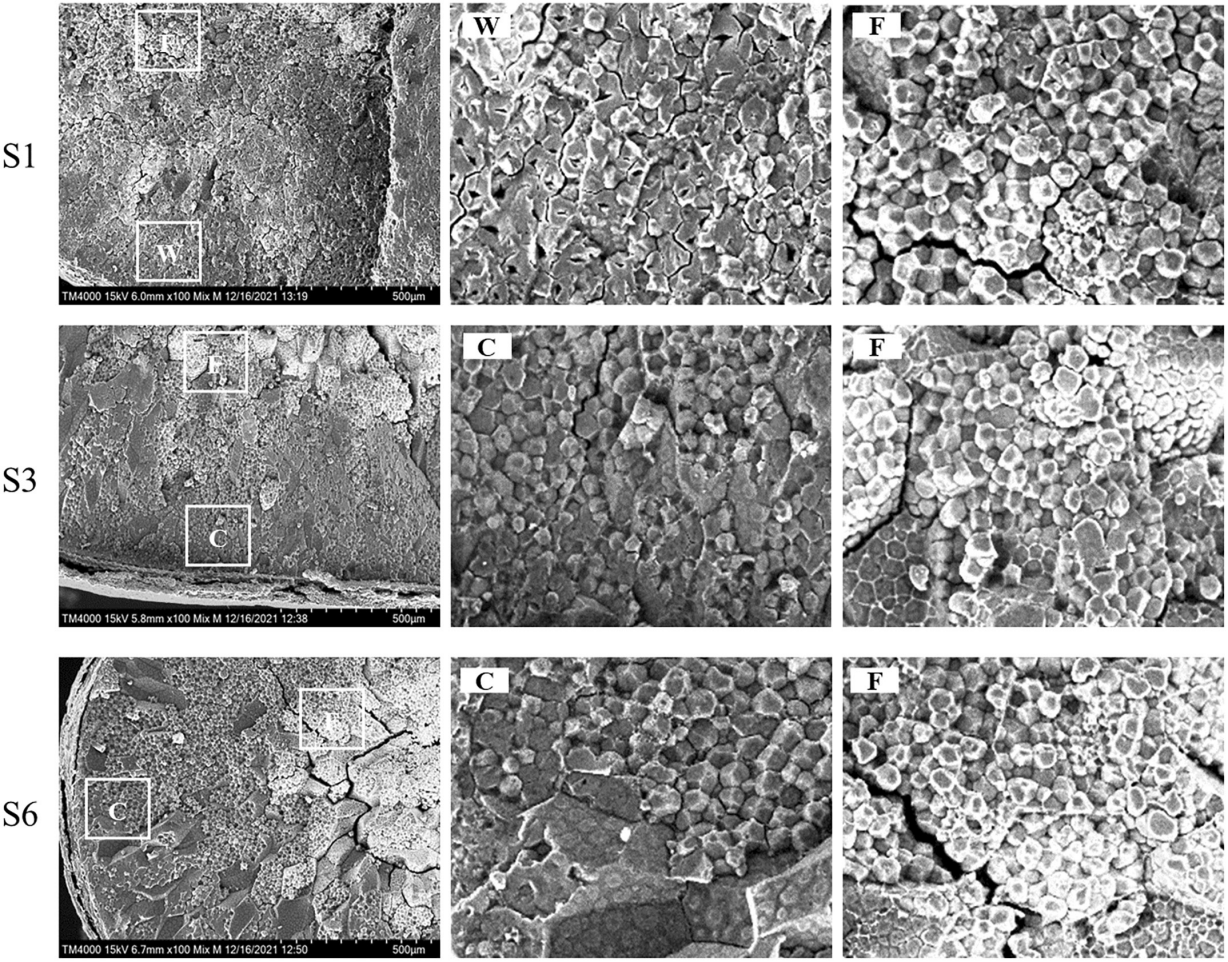
SEM micrographs of sorghum endosperm architecture. F refers to floury endosperm, W refers to waxy endosperm, and C refers to corneous endosperm.

Three sorghum grains with low (S1), intermediate (S3), and high (S6) amylose content were used to examine endosperm architecture by SEM (Figure 2). At a higher magnification, the outer layer endosperm was more compact than floury endosperm (F) in all sorghum grains. The waxy endosperm region (W) was more loosely packed than corneous endosperm (C). Further observation of the internal structure of starch granules in the W region showed that spindle‐shaped holes were present. However, there were no obvious cavities in starch granules of the C region. This is consistent with the result reported by Sattler et al. (2009). Chen et al. (2012) also found cavities in waxy maize starch granules. But the mechanism of hole formation is not clear and needs further study. The results indicated that the amylose content might affect the morphology of starch granules and then change the endosperm texture of sorghum grain.

#### 3.2.2 Starch granular morphology

**FIGURE 3:**
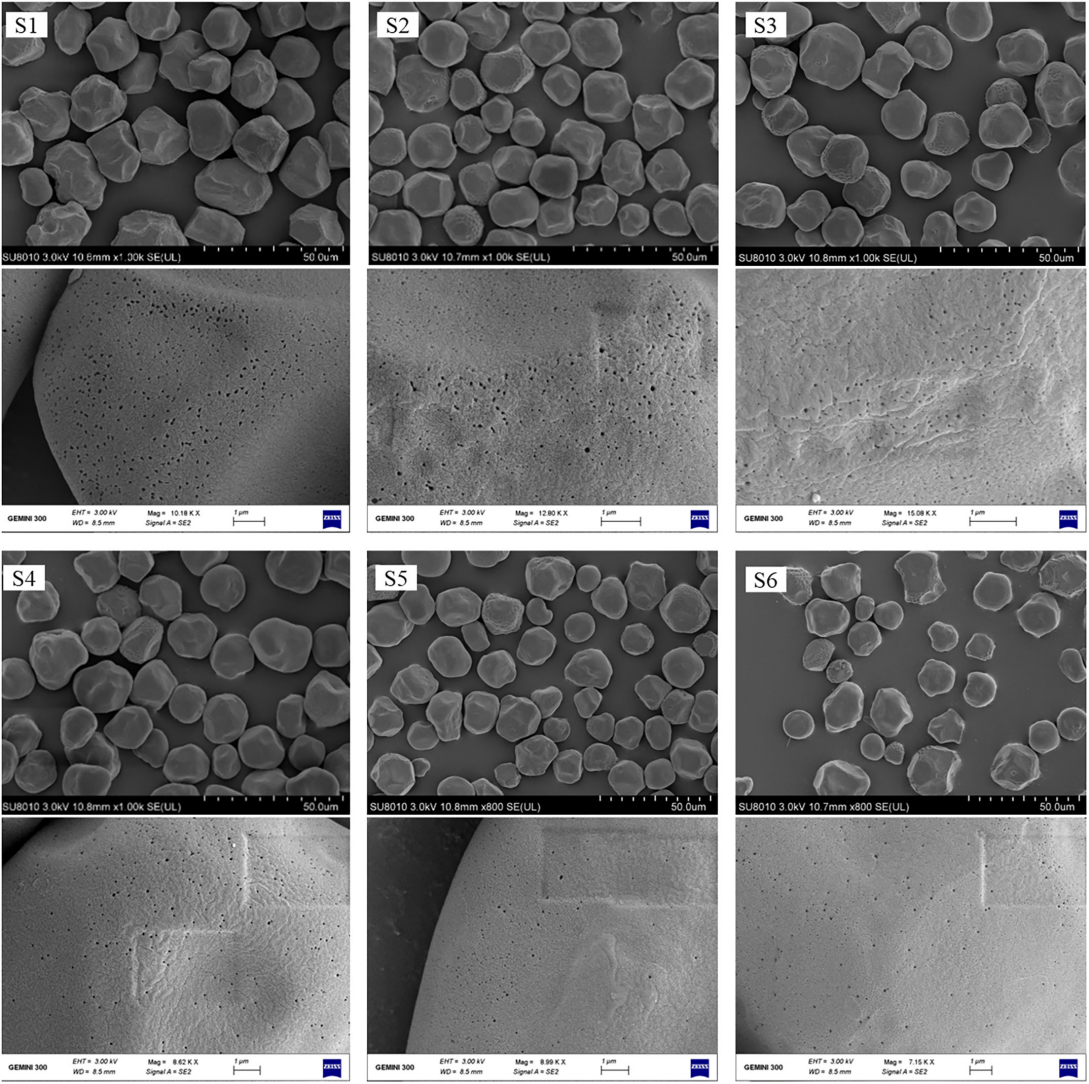
SEM micrographs of starch isolated from different sorghum grains.

SEM was used to reveal details of the granule surface (Figure 3). All starch granules of six sorghum varieties were mainly spherical, and the surface of some granules presented dents. At a higher magnification, we could find more and larger pinholes on the surface of starch granules from low‐amylose sorghum grains (S1 and S2) than those of other varieties. According to research reports, sorghum starch granules had tube‐like channels that penetrated from the external surface inward toward a cavity (Huber & Bemiller, 2000). The starch granules from the majority of varieties, such as barley (Sun et al., 2022), triticale, and corn (Naguleswaran et al., 2011), also had pinholes. The porous structure might contribute to the hydrolysis of the starch (Ai et al., 2011), suggesting that low‐amylose sorghum grains might have higher starch digestibility. However, the reasons why low‐amylose starch granules have more holes need to be further investigated.

**FIGURE 4:**
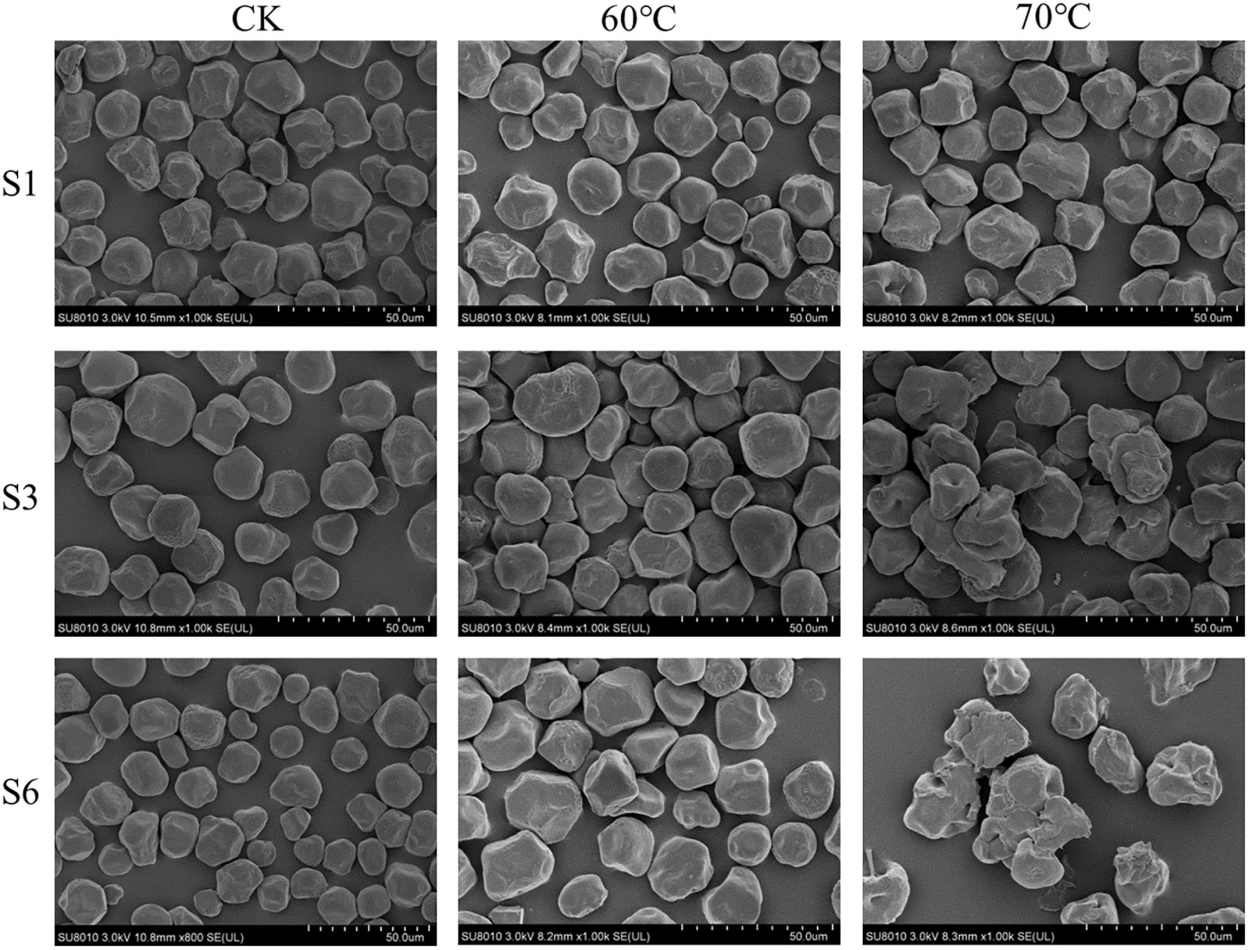
Scanning electron micrographs of heat‐treated starch isolated from different sorghum grains.

To verify the thermal stability of starch granules, the micro‐morphological changes of cooked starch were observed by SEM (Figure 4). All the untreated sorghum starch granules were essentially spherical. The starch granules treated at 60°C appeared to absorb water and produced a certain volume expansion, remained intact, in granular form. However, when the heat‐treatment temperature was up to 70°C, a small fraction of the intermediate‐amylose starch granules (S3) appeared fractured and swollen, lost the granular morphology of native starch, and agglomerated to form larger amorphous particles. And more extensive aggregation, depression, and serious deformation of high‐amylose starch granules (S6) could be observed. In conclusion, high‐amylose starch granules were more likely to absorb water and deform after heated, and eventually lost the granular morphology. The results showed that amylose content might be adverse to the thermal stability of sorghum starch.

#### 3.2.3 Molecular fine structure

The particle size distributions of all starch samples are presented in Figure S1. All particle size distributions displayed a unimodal distribution. As shown in Table 2, the average diameter of sorghum starch granules ranged from 18.48 μm to 20.55 μm. The granular size of sorghum starches showed a negative linear correlation (*r* = −0.982**) with their amylose contents as shown in Figure S2. Moreover, previous studies recommended that the granules of high‐amylose barley starches were smaller than low‐amylose barley starches (Stevneb et al., 2006). According to the reports, starch granule size might significantly affect thermal and gelatinization characteristics (Lin et al., 2016), digestibility (Noda et al., 2010), and crystallization level (Cai et al., 2014). This led us to speculate that the amylose content might affect functional properties of sorghum starch by affecting particle size.

**TABLE 2:** Molecular fine structure of starches isolated from different sorghum grains.

Sample	Granular diameter (μm)	Branch chain‐length distribution (%)			
		DP 6–12	DP 13–24	DP 25–36	DP > 36
S1	20.55 ± 0.00a	15.04 ± 0.43a	44.42 ± 0.22a	16.16 ± 0.29a	24.21 ± 0.41a
S2	20.09 ± 0.02ab	14.87 ± 0.32ab	44.34 ± 0.22a	16.18 ± 0.32a	24.44 ± 0.18a
S3	19.17 ± 0.01b	16.18 ± 0.34c	46.65 ± 0.20b	14.81 ± 0.16b	22.22 ± 0.30b
S4	18.68 ± 0.01c	16.06 ± 0.42c	46.53 ± 0.26b	14.93 ± 0.24b	22.37 ± 0.45b
S5	18.57 ± 0.02c	15.94 ± 0.37c	46.59 ± 0.38b	14.76 ± 0.25bc	22.60 ± 0.34bc
S6	18.48 ± 0.01c	16.22 ± 0.42c	46.66 ± 0.35b	14.74 ± 0.29bc	22.27 ± 0.40b

*Note*: Different lowercase letters on the shoulders of the same indicator indicate significant differences (*p* < .05).

According to a previous report, the weight‐based chain‐length distribution of amylopectin could be divided into four groups of fractions (Hanashiro et al., 1996). As shown in Table 2, the most abundant chain of the amylopectin appeared at DP 13–24 (DP, degree of polymerization). Similar results were reported in the study of maize (Lin et al., 2022), rice (Luo et al., 2021), wheat starches (Zi et al., 2019), and mung bean (Huong et al., 2021). Similar to the findings by Sang et al. (2008), the starches of S1 and S2 with low‐amylose content had a slightly higher proportion of chains with DP 25–36 (16.16% and 16.18%) and > 36 (24.21% and 24.44%) compared with other varieties. The correlative analysis exhibited that the amylose content was positively correlated to the proportion of the short branch chains (DP = 6–12 and 13–24, *r* = 0.913* and *r* = 0.948**) but negatively correlated to the proportion of the long branch chains (DP = 25–36 and >36, *r* = −0.954** and *r* = −0.919**) (Figure S3). Thus, we concluded that the fine structure of sorghum starch had strong correlations with amylose content.

#### 3.2.4 Crystalline structure

**FIGURE 5:**
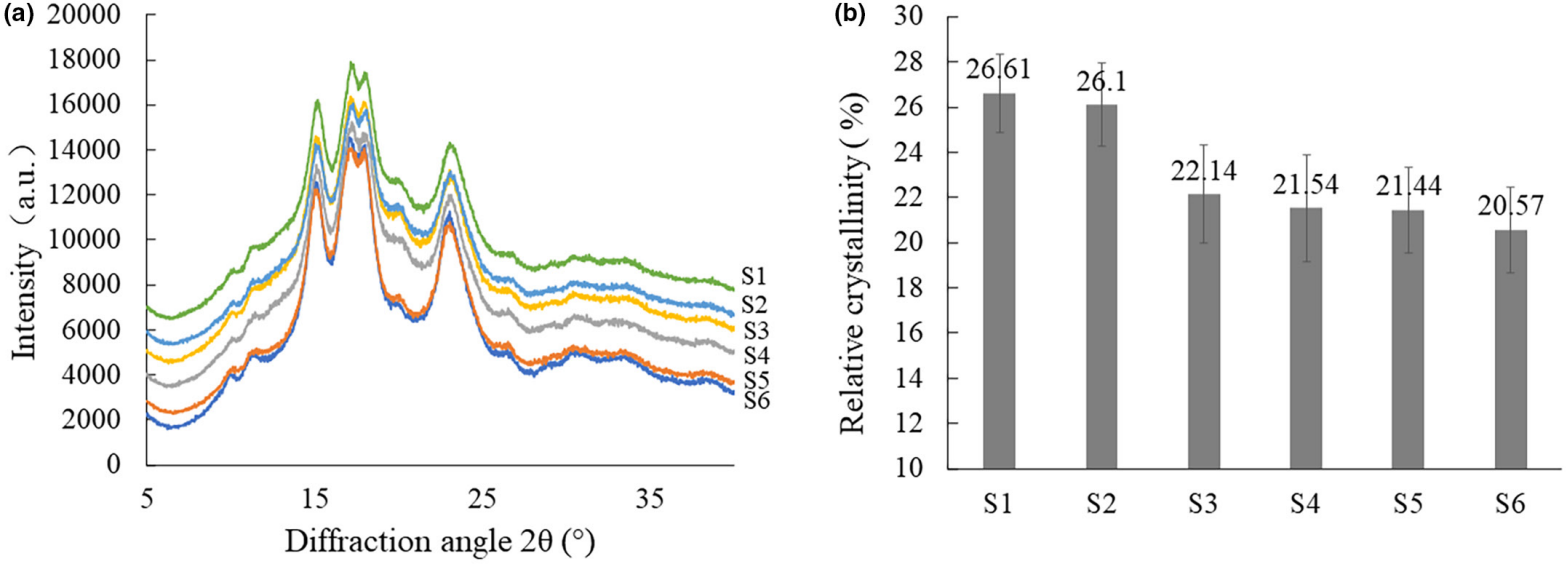
X‐ray diffraction patterns (a) and relative crystallinity (b) of starches isolated from different sorghum grains.

According to the XRD patterns (Figure 5a), all the starches showed typical A‐type diffraction pattern, similar to the findings of Mohamed et al. (2016) with major peaks at 15.2° 2θ, 17.2° 2θ, 17.8° 2θ, and 22.9° 2θ. As shown in Figure 5b, the starches of S1 and S2 with low‐amylose content exhibited the highest degree of crystallinity (26.61% and 26.10%), whereas the high‐amylose sorghum starches (S5 and S6) showed the lowest degree of crystallinity (21.44% and 20.57%). There was a significant negative correlation between the relative crystallinity and amylose content (*r* = −0.975**) (Figure S4). Similar to the findings by Singh et al. (2017), amylose would decrease the crystallinity of the starch granules. The previous research reported that the amylopectin in the form of double helices contributed predominantly to formation of crystalline structure, but amylose could significantly inhibit the formation of ordered structure in sorghum starch granules, thus decreasing the relative crystallinity (Creek et al., 2006; Lopez‐Rubio et al., 2010).

### 3.3 Physicochemical properties

#### 3.3.1 Gelatinization properties

According to Table 3, DSC analysis exhibited that the starches of S1 and S2 showed a highest gelatinization temperatures (*T*_*o*_, *T*_*p*_, and *T*_*c*_) and *ΔH*, indicating the higher temperature and more energy required for gelatinization of low‐amylose sorghum grains. The amylose content showed negative linear correlations with *T*_*o*_ and *T*_*p*_ (*r* = −0.894* and *r* = −0.914*, respectively) (Figure S5). In other words, the higher the amylose content of the starches, the lower the gelatinization transition temperatures. Trust et al. (2001) also found a significant correlation between amylose content and *T*_*p*_. However, there was no significant correlation between amylose content and *T*_*c*_. During gelatinization, starch granules gradually absorb water and expand, and then semi‐crystalline structure of starch is destroyed (Cooke & Gidley, 1992). Therefore, low‐amylose starches which exhibited a high relative crystallinity required more energy to destroy the crystalline structure, indicating higher *T*_*o*_ and *T*_*p*_. There was a significant negative linear correlation between *ΔH* and amylose content (*r* = −0.975**) (Figure S5). Thus, low‐amylose starches required more enthalpy to completely gelatinize them, indicating a better thermal stability. The DSC analysis results were also in agreement with the phenomenon observed by SEM (Figure 4).

**TABLE 3:** Thermal properties of starches isolated from different sorghum grains.

Sample	*T*_*o*_ (°C)	*T*_*p*_ (°C)	*T*_*c*_ (°C)	Δ*H* (J/g)
S1	74.47 ± 0.17a	78.15 ± 0.30a	84.94 ± 1.14ab	14.28 ± 2.98a
S2	74.73 ± 0.15a	78.40 ± 0.29a	84.86 ± 0.85ab	13.13 ± 2.09ab
S3	71.52 ± 0.13b	75.13 ± 0.21b	81.52 ± 1.01cd	11.32 ± 1.72cd
S4	72.70 ± 0.21c	76.04 ± 0.42c	81.91 ± 0.1.3c	10.36 ± 0.41de
S5	72.12 ± 0.22d	75.56 ± 0.35bc	83.78 ± 0.32b	9.56 ± 1.07e
S6	71.54 ± 0.08b	74.76 ± 0.08d	80.31 ± 0.35d	9.80 ± 0.39e

*Note*: *T*_*o*_, *T*_*p*_, *T*_*c*_ represent onset, peak, and conclusion temperatures, respectively, and Δ*H* represents enthalpy of gelatinization. Different lowercase letters on the shoulders of the same indicator indicate significant differences (*p* < .05).

#### 3.3.2 Pasting properties

As shown in Table 4, the starches of S1 and S2 showed the highest peak viscosity (PV) of 2634 cp and 2413 cp and hot paste viscosity (HPV) of 1693 cp and 1536 cp, respectively. Moreover, the starches of S1 and S2 also exhibited the lowest final viscosity (FV) of 2299 cp and 2292 cp and setback (SB) of 606 cp and 756 cp, respectively, indicating that the low‐amylose sorghum starches exhibited greater resistance against retrogradation during cooling. Our observation is similar to that of Sang et al. (2008) who found waxy sorghum starch showed a lower pasting temperature and higher PV than higher amylose sorghum starch. It is due to the fact that low‐amylose sorghum starches have higher relative crystallinity and greater amounts of long chains (Jane et al., 1999). Amylose content had a negative linear correlation with PV (*r* = −0.856*) and positive linear correlations with FV and SB (*r* = 0.890* and *r* = 0.983**, respectively) but no significant correlations with HPV and breakdown (BD) (Figure S6).

**TABLE 4:** Pasting properties of starches isolated from different sorghum grains.

Sample	PV (cp)	HPV (cp)	BD (cp)	FV (cp)	SB (cp)
S1	2634 ± 16.33^a^	1693 ± 35.67^a^	941 ± 16.55^a^	2299 ± 18.76^a^	606 ± 11.23^a^
S2	2413 ± 44.23^b^	1536 ± 20.35^a^	877 ± 22.36^b^	2292 ± 36.36^a^	756 ± 21.36^a^
S3	2223 ± 37.65^cd^	1352 ± 36.47^b^	871 ± 13.54^b^	2692 ± 23.89^b^	1340 ± 15.68^b^
S4	2209 ± 53.28^c^	1302 ± 56.32^b^	907 ± 28.33^ab^	2637 ± 13.33^b^	1335 ± 45.65^b^
S5	2193 ± 32.35^c^	1413 ± 34.25^b^	780 ± 31.25^c^	2765 ± 23.58^c^	1352 ± 45.63^b^
S6	2385 ± 39.56^d^	1511 ± 31.58^a^	874 ± 17.36^b^	3027 ± 48.69^d^	1516 ± 36.25^c^

*Note*: Different lowercase letters on the shoulders of the same indicator indicate significant differences (*p* < .05).

Abbreviations: BD, break down; FV, final viscosity; HPV, hot paste viscosity; PV, peak viscosity; SB, setback.

#### 3.3.3 In vitro starch digestibility

According to Table 5, it was observed that the RDS, SDS, and RS contents of starches from six sorghum varieties were in the ranges of 89.89–93.11%, 3.61–7.37%, and 2.56–3.39%, respectively (Table 5). The low‐amylose starches (S1 and S2) exhibited the lowest RDS (89.89% and 91.24%, respectively) and RS (2.74% and 2.56%, respectively) and the highest SDS (7.37% and 6.20%, respectively). Moreover, the amylose content was positively correlated to RDS (*r* = 0.853*), and negatively correlated to SDS (*r* = −0.849*) (Figure S7). Shirmohammadi et al. (2021) found that the holes on the surface of starch granules contributed to enhance the degradability of barley grain. Therefore, the low‐amylose starch granules could be easily accessed by the digestive enzymes because of the more porous structure (Figure 3), leading to lower RS. Although RS of low‐amylose sorghums used in this study is slightly lower than that of previously reported waxy sorghum (8.4%) (Sang et al., 2008), it is notable that solid‐state fermentation of sorghum mainly consumed RDS and SDS instead of RS irrespective of sorghum variety (Xu et al., 2021). Therefore, it is inferable that the sorghum varieties with low‐amylose content were desirable for high ethanol yield.

**TABLE 5:** The RDS, SDS, and RS contents of starches isolated from different sorghum grains.

Sample	RDS (%)	SDS (%)	RS (%)
S1	89.89 ± 1.10a	7.37 ± 0.63a	2.74 ± 0.85bc
S2	91.24 ± 0.78b	6.20 ± 0.34b	2.56 ± 0.49c
S3	93.03 ± 11.28c	3.61 ± 0.52c	3.37 ± 0.83ab
S4	91.25 ± 3.37b	5.92 ± 0.59b	2.83 ± 0.76bc
S5	93.11 ± 5.86c	3.77 ± 0.31c	3.12 ± 0.36bc
S6	92.96 ± 3.24c	3.65 ± 0.14c	3.39 ± 0.25ab

*Note*: Different lowercase letters on the shoulders of the same indicator indicate significant differences (*p* < .05).

## 4 CONCLUSIONS

In our study, we found that amylose contents of six sorghum grains ranged from 0.2% to 21.3%. The SEM indicated that the endosperm texture of low‐amylose sorghum was waxy, but the intermediate‐amylose and high‐amylose sorghum were corneous and its starch granules were more compactly packed. There were spindle‐shaped holes in the starch granules of the waxy endosperm region. But no obvious holes were found in the starch granules of the corneous endosperm region. The starches with higher amylose content exhibited fewer micropores on the surface of starch granules, and were less accessible to the digestive enzymes, resulting in lower starch digestibility. The higher amylose starch granules had more defective crystalline structure, smaller particle size, and lower amounts of long chains, leading to a poor thermal stability and resistance against retrogradation. Our findings suggested that amylose content had strong correlations with physicochemical properties and granular structure of sorghum starch and endosperm texture of sorghum grains. It could be speculated that sorghum grains with low‐amylose content might have higher starch digestibility because of reduced kernel density, the increased space between starch granules (Figure 2) and more porous structure of starch granules (Figure 3), resulting in higher ethanol yield and fermentation efficiency. The results indicated that sorghum varieties with lower amylose content might have a great potential to be an excellent raw material for baijiu. In conclusion, this study clarified the relationships between amylose content and sorghum starch properties, thus providing references to choose appropriate raw materials for baijiu production. However, the further research on the association between starch properties and the fermentation performance of more sorghum varieties is necessary.

## AUTHOR CONTRIBUTIONS

**Longping Yang:** Conceptualization (equal); data curation (equal); formal analysis (equal); investigation (equal); writing – original draft (equal); writing – review and editing (equal). **Lei Li:** Data curation (equal); formal analysis (equal); methodology (equal); writing – review and editing (equal). **Feng Jiang:** Data curation (equal); methodology (equal); project administration (equal); writing – original draft (equal). **Qiaoling Zhang:** Data curation (equal); supervision (equal); visualization (equal). **Fan Yang:** Conceptualization (equal); project administration (equal). **Yilin Wang:** Formal analysis (equal); methodology (equal). **Zhenyu Zhao:** Resources (equal); supervision (equal). **Qingfan Ren:** Methodology (equal); writing – review and editing (equal). **Li Wang:** Funding acquisition (equal); resources (equal); supervision (equal).

## FUNDING INFORMATION

This work received funds from Kweichow Moutai Co., Ltd. (Grant No. 2018006).

## CONFLICT OF INTEREST STATEMENT

The authors declare that they do not have any conflict of interest.

## Supporting information


Figure S1.

## Data Availability

Research data are not shared.
