# Supplementary material for: Structural and physicochemical characteristics of starches from sorghum varieties with varying amylose content
Source: Food Sci Nutr. 2024 Aug 20;12(10):7989–99. doi: 10.1002/fsn3.4245 (PMC11521702; doi:10.1002/fsn3.4245)
Supplement: Supplementary file 1 — Figure S1. [file FSN3-12-7989-s001.docx]

**Supplementary Materials:** Supporting information may be found in the online version of this article (Figures S1, S2, S3, S4, S5, S6 and S7)


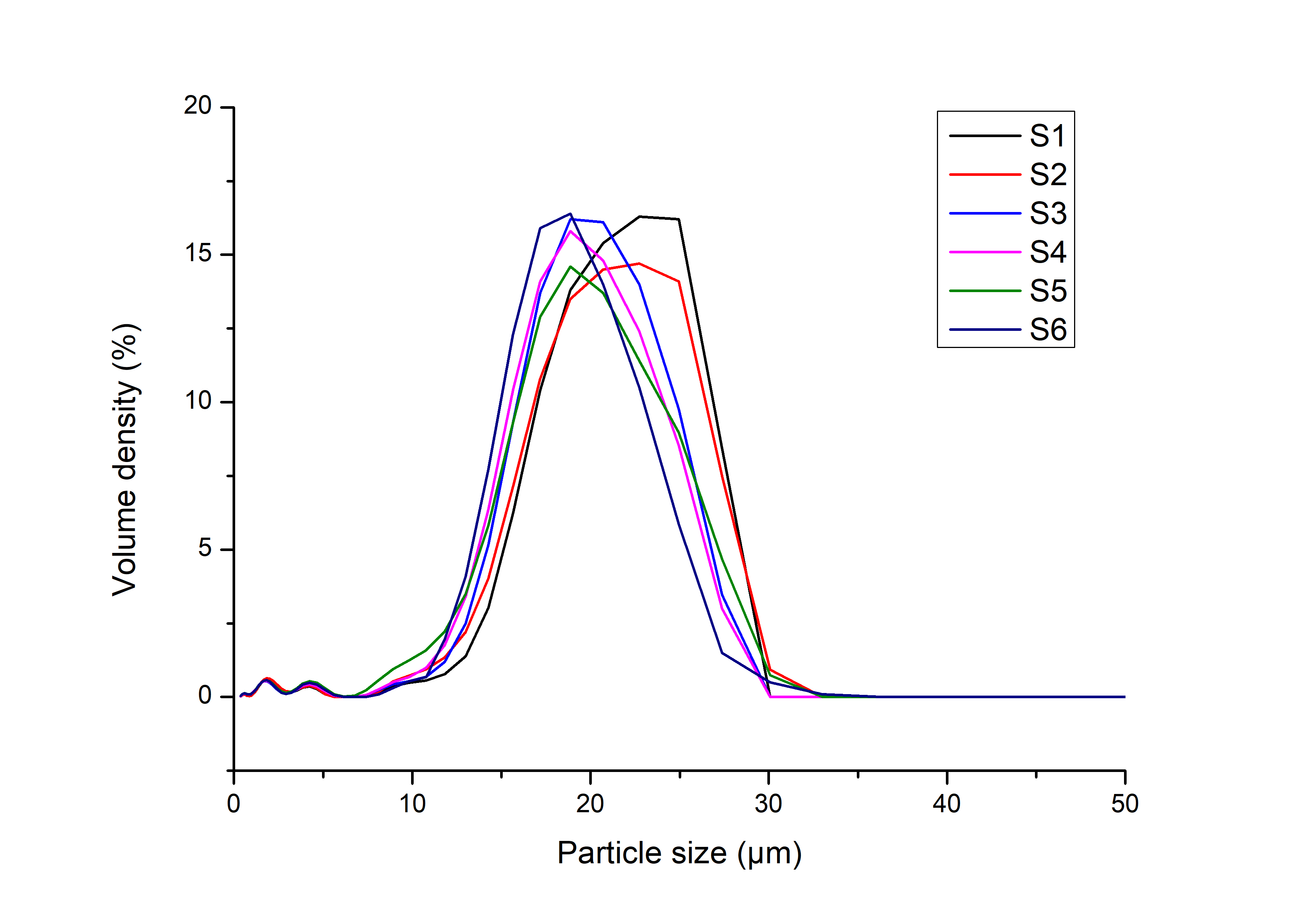


Figure S1. Particle size distributions of six sorghum starches.

Figure S2. Correlation coefficients between amylose content and the granular size of sorghum starches.


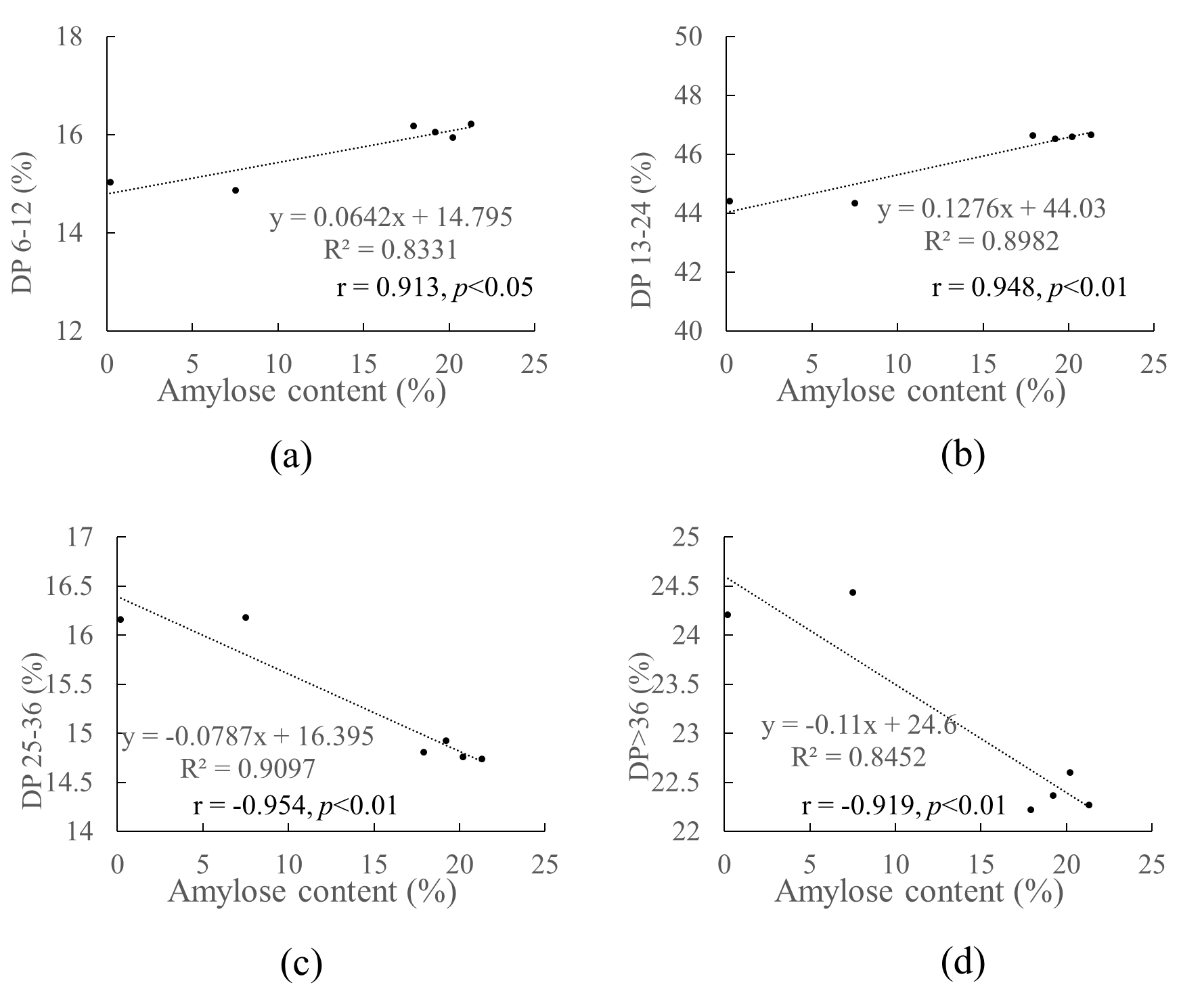


Figure S3. Correlation coefficients between amylose content and the chain-length distribution parameters.

Figure S4. Correlation coefficients between amylose content and relative crystallinity.

Figure S5. Correlation coefficients between amylose content and gelatinization properties.


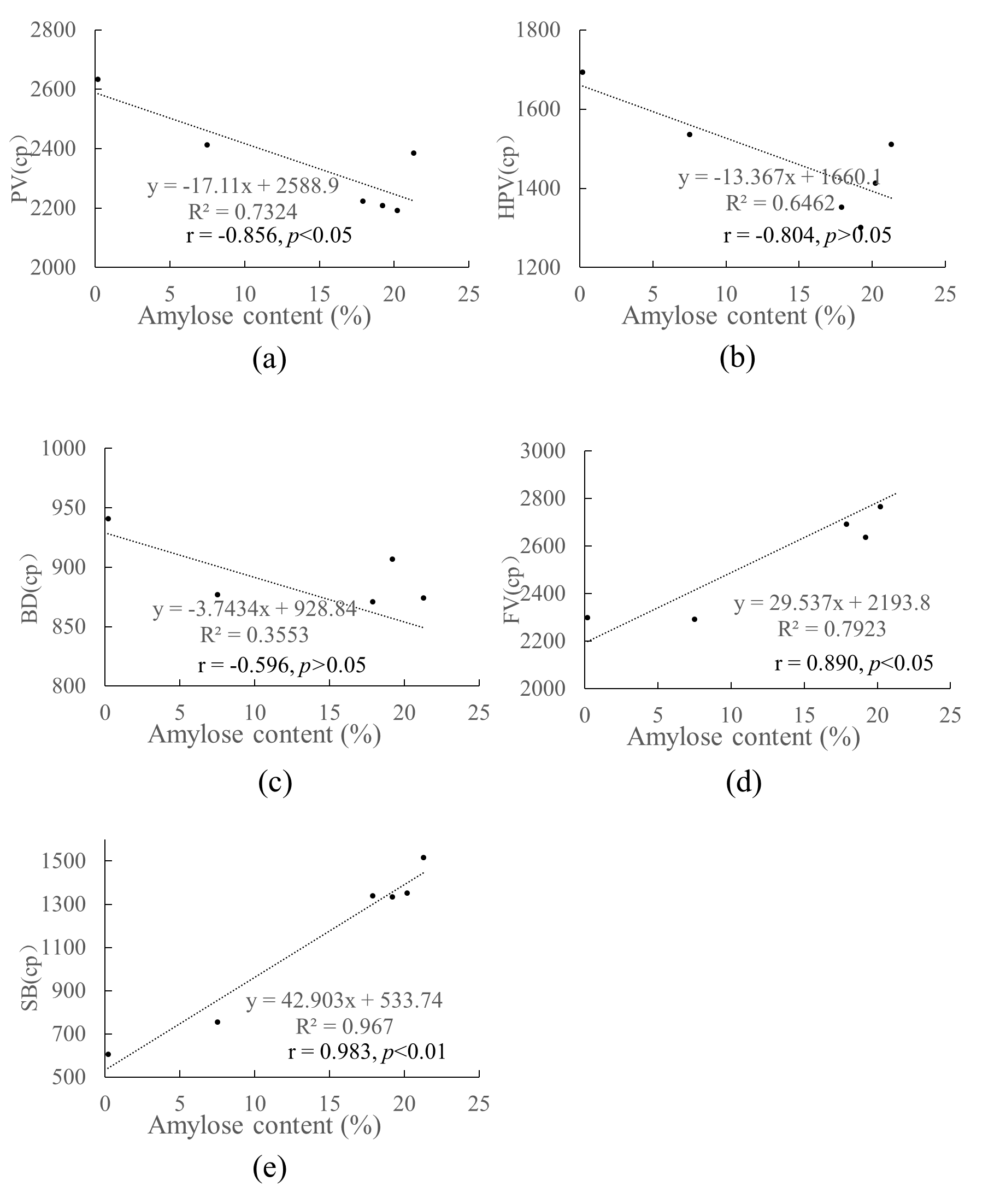


Figure S6. Correlation coefficients between amylose content and pasting properties.

Figure S7. Correlation coefficients between amylose content and in vitro starch digestibility.
